# Antiphotoaging and Antimelanogenic Effects of *Penthorum chinense* Pursh Ethanol Extract due to Antioxidant- and Autophagy-Inducing Properties

**DOI:** 10.1155/2019/9679731

**Published:** 2019-04-03

**Authors:** Deok Jeong, Jongsung Lee, Sang Hee Park, You Ah Kim, Byoung Jun Park, Junsang Oh, Gi-Ho Sung, Adithan Aravinthan, Jong-Hoon Kim, Hakhee Kang, Jae Youl Cho

**Affiliations:** ^1^Department of Integrative Biotechnology, Sungkyunkwan University, Suwon, Republic of Korea; ^2^Department of Biocosmetics, Sungkyunkwan University, Suwon, Republic of Korea; ^3^Skin Science Research Institute, Kolmar Korea Co. Ltd., Chungcheongbuk-do 28116, Republic of Korea; ^4^Institute for Healthcare and Life Science, International St. Mary's Hospital and College of Medicine, Catholic Kwandong University, Incheon 22711, Republic of Korea; ^5^Department of Physiology, College of Veterinary Medicine, Chonbuk National University, Iksan, Republic of Korea

## Abstract

*Ethnopharmacological Relevance. Penthorum chinense* Pursh (Penthoraceae) is a traditional herbal plant that has been used in China for the treatment of jaundice, cholecystitis, edema, and infectious hepatitis. In addition, the Korea Medicinal Plant Dictionary states that *Penthorum chinense* Pursh can be used to treat contusions and skin bruises by improving blood flow. Recent studies have shown that *Penthorum chinense* Pursh ethanol extract (Pc-EE) exhibits strong antioxidant effects. In this study, we examined the effects of Pc-EE on UVB-induced or H_2_O_2_-induced oxidative stress, as well as its antimelanogenic properties. Cell viability, matrix metalloproteinase (MMP) expression, cyclooxygenease-2 (COX-2), and interleukin-6 (IL-6) expression and moisturizing factors were investigated in keratinocytes. Collagen synthesis induction was measured in HEK293T cells. For melanogenesis, the effects of Pc-EE on melanin content and tyrosinase activity were measured. Additionally, the antimelanogenic- and autophagy-inducing activities of Pc-EE were examined using immunoblotting and confocal microscopy. Pc-EE protected HaCaT cells against death from UVB irradiation- or H_2_O_2_-induced oxidative stress. Pc-EE increased the promoter activity of the type 1 procollagen gene Col1A1 and decreased the expression of MMPs, COX-2, IL-6, and hyaluronidase induced by UVB irradiation- or H_2_O_2_-induced oxidative stress. Pc-EE showed a strong antioxidant effect in the DPPH assay. In *α*-melanocyte-stimulating hormone- (*α*-MSH-) stimulated B16F10 cells, Pc-EE reduced melanin production, decreased tyrosinase expression and microphthalmia-associated transcription factor (MITF) protein levels, and decreased the phosphorylation levels of p38 and JNK. In HEK293T cells, Pc-EE promoted the expression of GFP-LC3B. In B16F10 cells, the LC3B and melanin contents were reduced by Pc-EE and were restored by the autophagy inhibitor 3-methyladenine (3-MA). These results suggest that Pc-EE can be used as a skin protection agent due to its antiapoptotic, antiaging, anti-inflammatory, and antimelanogenic properties.

## 1. Introduction

The skin plays an important role in protecting our bodies from the environment and is the first organ to be exposed to external stresses such as ultraviolet (UV) radiation, infectious pathogens, and hazardous chemicals. These various external stimuli induce oxidative stress in the skin, leading to biological reactions such as inflammation induction, melanogenesis, photoaging, and skin cancer [1–4]. UV radiation is one of the most important risk factors for inducing oxidative stress and is divided into three categories according to wavelength: UVA (315-400 nm), UVB (280-315 nm), and UVC (100-280 nm). Among these, UVB is more genotoxic and causes more sunburns compared with the other wavelengths [5]. Oxidative stress induced by UVB irradiation in skin cells induces a variety of biological responses. In particular, reactive oxygen species (ROS) such as superoxide anion, hydrogen peroxide, and hydroxyl radicals produced by exposure to UV irradiation induce DNA mutations, resulting in about 65% of melanomas and about 90% of nonmelanoma skin cancers [6]. In addition, UVB-induced oxidative stress can increase matrix metalloproteinases (MMPs), which degrade extracellular matrix components, such as collagen, and contribute to wrinkle formation by keratinocytes [1, 7, 8]. Also, oxidative stress induced by UVB irradiation activates the nuclear factor- (NF-) *κ*B signaling pathway, induces cyclooxygenease-2 (COX-2) and interleukin-6 (IL-6) expression, and induces inflammation in keratinocytes [2]. Hyaluronic acid (HA), one of the polysaccharide groups found in connective tissue and the epithelium, is one of the major components of the extracellular matrix and maintains the extracellular space of the epidermis in dermal tissue [9, 10]. UVB irradiation induces hyaluronidase [11], a degradative enzyme of HA, to promote skin aging through loss of skin moisture and laxity [12]. In addition, UV irradiation induces p53 activation and induces *α*-melanocyte-stimulating hormone (*α*-MSH) in keratinocytes [3]. The induced *α*-MSH is secreted and binds to the melanocortin 1 receptor (MC1R) of melanocytes, activating signal transduction and inducing the microphthalmia-associated transcription factor (MITF). The activity of MITF is regulated by mitogen-activated protein kinases (MAPKs), and the activated MITF induces tyrosinase, TYRP1, and TYRP2 to produce melanin [13]. Although UVB-induced melanogenesis is a well-known reason why we do develop photoprotective materials for cosmetic purposes [14], altering cellular metabolism and generating genotoxic and mutagenic properties by oxidative stress causing skin cancer are also big issues in terms of the skin toxicological aspect [15]. Therefore, the importance of photoprotection from UV irradiation is considered in view of not only the regulation of biological responses such as wrinkling, inflammation, and melanogenesis of skin cells but also the prevention of skin cancers to maintain skin health conditions.

*Penthorum chinense* Pursh is distributed in East Asian countries, including Korea, China, and Japan, and is traditionally used as a medicinal herb or tea. In particular, it has been used in China for the treatment of jaundice, cholecystitis, edema, and infectious hepatitis [16]. In addition, the Korea Medicinal Plant Dictionary states that *Penthorum chinense* Pursh can be used to treat contusions by improving blood flow. *Penthorum chinense* Pursh ethanol extract (Pc-EE) exerts a strong antioxidant effect in ethanol-induced liver injury [16] and tert-butyl hydroperoxide- (*t*-BHP-) induced liver cell damage [17]. Recent studies have also shown that *Penthorum chinense* Pursh inhibits the production and expression of inflammatory mediators and cytokines such as nitric oxide (NO), tumor necrosis factor-alpha (TNF-*α*), and interleukin-1*β* (IL-1*β*) in lipopolysaccharide- (LPS-) induced macrophage-mediated inflammatory responses, implying its excellent anti-inflammatory effects [18]. Despite these excellent antioxidant and anti-inflammatory effects, the pharmacological role of *Penthorum chinense* Pursh in the biological response of skin cells under UVB- or H_2_O_2_-induced condition has not been fully elucidated.

Therefore, in this study, we looked over the effect of *Penthorum chinense* Pursh ethanol extract (Pc-EE) with antioxidative and anti-inflammatory properties by using keratinocyte cell line HaCaT cells. Particularly, we found protective effects on UVB irradiation- or H_2_O_2_-induced responses especially in cell death, skin tissue remodeling, water loss, inflammation, and ROS production. Moreover, we have newly demonstrated the molecular mechanisms of Pc-EE-mediated antimelanogenesis by using the melanoma cell line B16F10.

## 2. Materials and Methods

### 2.1. Materials

95% ethanol extract of *Penthorum chinense* Pursh was donated by the National Institute of Biological Resources (https://www.nibr.go.kr/, Incheon, Korea). 3-(4-5-Dimethylthiazol-2-yl)-2-5-diphenyltetrazolium bromide (MTT), 5-hydroxy-2-(hydroxymethyl)-4H-pyran-4-one (kojic acid), monophenol monooxygenase (mushroom tyrosinase), 4-hydroxyphenyl-*β*-D-glucopyranoside (arbutin), 1,1-diphenyl-2-picrylhydrazyl (DPPH), *α*-MSH, L-DOPA ethyl ester, ascorbic acid, and forskolin were purchased from Sigma-Aldrich (St. Louis, MO, USA). Luciferase plasmids harboring promoter binding sites for CREB and Col1A1 were used as reported earlier [19]. TRIzol reagent was obtained from Molecular Research Center (Montgomery, OH, USA). Fetal bovine serum and Dulbecco's modified Eagle's media (DMEM) were purchased from Gibco (Grand Island, NY, USA). The cell lines used in the present experiments (HEK293T, HaCaT, and B16F10 cells) were obtained from ATCC (Rockville, MD, USA). Total and phospho-specific antibodies of CREB (#9198), phospho-CREB (#9198), lamin A/C (#4777), *β*-actin (#4967), ERK (#4696), phospho-ERK (#9101), p38 (#9212), phospho-p38 (#4631), JNK (#4672), phospho-JNK (#9255), and LC3B (#2775) were purchased from Cell Signaling Technology (Beverly, MA, USA). MITF (#71588) and tyrosinase (#73244) were purchased from Santa Cruz Biotechnology (Dallas, Texas, USA). All other chemicals were obtained from Sigma-Aldrich (St. Louis, MO, USA).

### 2.2. Cell Culture

B16F10 (a mouse melanoma cell line) and HaCaT cells (a human keratinocyte cell line) were cultured in DMEM supplemented with 10% fetal bovine serum and 1% antibiotics (penicillin and streptomycin). HEK293T cells (a human embryonic kidney cell line) were cultured in DMEM supplemented with 5% fetal bovine serum and 1% antibiotics. All cell lines were cultured in a CO_2_ incubator (5%) at 37°C.

### 2.3. Cell Viability Assay

B16F10 and HEK293T were cultured in 96-well plates at a density of 2.5 × 10^4^ or 6.25 × 10^4^ cells/well, respectively, and HaCaT cells were seeded onto 24-well plates at a density of 2.5 × 10^5^ cells/well. All cell lines were cultured in fresh complete culture medium. To determine the cytotoxicity of Pc-EE, cells were treated with 25, 50, 100, or 200 *μ*g/mL Pc-EE in each experimental condition. To test the effects of Pc-EE on UVB- or H_2_O_2_-induced toxicity in HaCaT cells, the cells were treated with either 30 mJ/cm^2^ UVB or 50 mM H_2_O_2_ and cultured in complete culture medium with 50, 100, or 200 *μ*g/mL Pc-EE for 24 h. Cell viability was determined using a conventional MTT assay [20]. The cell viability was expressed as a percent using the following formula: cell viability (%) = [A0/A1] × 100, where A0 is the absorbance of the sample mean value and A1 is the absorbance of the normal group mean value.

The PI-staining experiment was also employed to confirm the effect of Pc-EE on the viability of HaCaT cells. Briefly, HaCaT cells (5 × 10^5^ cells/mL) were plated in 12-well plates and incubated overnight. Pc-EE (0-100 *μ*g/mL) was then added to the cells at the indicated doses. After 24 h, the cells were harvested, washed twice with PBS, and resuspended in 1x binding buffer. PI (50 *μ*g/ml) was applied to the cells, which were then incubated for 15 min at room temperature in the dark. Fluorescence signals from the cells were measured using a BD FACScan flow cytometer (Becton Dickinson, Mountain View, CA, USA) and CellQuest Pro (IVD) software (Becton Dickinson).

### 2.4. DPPH Decolorimetric Assay

To confirm the antioxidant effects of Pc-EE, a DPPH decolorimetric assay was performed. Either Pc-EE (25-100 *μ*g/mL) or ascorbic acid (50 *μ*M) was added to 495 *μ*L of DPPH (250 *μ*M in methanol) and incubated at 37°C for 30 min. After the reaction was complete, the absorbance of each fraction was measured at 517 nm using a spectrophotometer. The DPPH scavenging effect was expressed as percent inhibition using the following formula: DPPH scavenging effect (%) = [(A0 − A1)/A0] × 100%, where A0 is the absorbance of DPPH alone and A1 is the absorbance of the sample.

### 2.5. High-Performance Liquid Chromatography (HPLC)

To confirm the phytochemical characteristics of Pc-EE (50 mg/mL), high-performance liquid chromatography (HPLC) analysis by injecting 10 *μ*L of this extract was performed using the standard compounds quercetin, luteolin, and kaempferol. The HPLC system was equipped with a Knauer WellChrom K-1001 HPLC pump, a WellChrom K-2600 fast scanning spectrophotometer, and a 4-channel K-500 degasser. Elution solvent A was 0.1% H_3_PO_4_ in H_2_O, and solvent B was acetonitrile. The gradient step of the solvent was solvent A to solvent B/min, and a Phenomenex Gemini C18 ODS (5 *μ*m) column was used. The content of standard compounds (quercetin, luteolin, and kaempferol) was expressed as a percent using the following formula: the content (%) = [amount of standard compound (ppm, mg/L)/concentration of the sample (mg/mL)] × 100%. HPLC was performed as described previously [20].

### 2.6. UVB Irradiation

The UVB irradiation experiment was performed with HaCaT cells at 312 nm using a UVB lamp (Bio-Link BLX-312, Vilber Lourmat, France) as described previously [21].

### 2.7. Analysis of mRNA Levels Using Reverse Transcriptase-Polymerase Chain Reaction (RT-PCR)

To quantify the levels of mRNA expression, HaCaT or B16F10 cells were treated with UVB irradiation (30 mJ/cm^2^), H_2_O_2_ (50 *μ*M), or *α*-MSH (100 nM) together with Pc-EE (50 or 100 *μ*g/mL). Total RNA was then isolated using TRIzol reagent according to the manufacturer's instructions. The PCR reaction was performed using 2x PCRBIO HS Taq Premix (PCR Biosystems, UK) under these incubation conditions (an initial denaturation and enzyme activation time of 2 min at 94°C, a 15 s denaturation time at 94°C, an annealing time of 15 s at 55-60°C, an extension time of 30 s at 72°C, and a final extension of 5 min at 72°C at the end of 30 cycles). The primers used in this study are listed in Table 1.

### 2.8. Plasmid Transfection and Luciferase Reporter Gene Assay

For the luciferase reporter gene assay, HEK293T cells (1.0 × 10^5^ cells/well in 24-well plates) were transfected with 0.8 *μ*g/mL of plasmids driving the expression of *β*-galactosidase, CREB-Luc, or Col1A1-Luc. Cells were transfected using the polyethylenimine (PEI) method as reported previously [22] and incubated for 24 h. The HEK293T cells were then treated with either 50 or 100 *μ*g/mL Pc-EE or 50 *μ*g/mL retinol for a further 24 h. CREB-luciferase expression was induced using 200 nM forskolin for 24 h. The luciferase activity was expressed as a percent using the following formula: the gene expression level: A0/B0 = C0, where A0 is the absorbance of luciferin and B0 is the absorbance of *β*-galactosidase. The luciferase activity (%) = [mean value of C0/mean value of C1] × 100%, where C0 is the absorbance of the gene expression level and C1 is the absorbance of the control group.

### 2.9. Melanin-Formation Assay

For the melanin-formation assay, B16F10 cells (1.0 × 10^5^ cells/well in 12-well plates) treated with 100 nM *α*-MSH in the presence or absence of 50 or 100 *μ*g/mL of Pc-EE, 1 mM arbutin, or 4 mM 3-methyladenine (3-MA) for 48 h were used. Determination of the melanin content was carried out according to a previous report [21]. The melanin contents were expressed as a percent using the following formula: the melanin contents (%) = [A0/A1] × 100, where A0 is the absorbance of the sample mean value and A1 is the absorbance of the control group mean value.

### 2.10. Tyrosinase Assay

For the tyrosinase assay [21], 50 mL of 6 mM L-DOPA (dissolved in 50 mM potassium phosphate buffer, pH 6.8) was added to each well of a 96-well plate. Next, 50 *μ*L of test compound (50, 100, or 200 *μ*g/mL Pc-EE or 300 *μ*M kojic acid dissolved in potassium phosphate buffer) was added, and the cells were incubated at room temperature for 15 min. Mushroom tyrosinase (100 units/mL) dissolved in potassium phosphate buffer was then added to the mixture. The absorbance of the mixture at 475 nm was measured immediately using a multidetection microplate reader. The tyrosinase activity was expressed as a percent using the following formula: the tyrosinase activity (%) = [A0/A1] × 100, where A0 is the absorbance of the sample mean value and A1 is the absorbance of the control group mean value.

### 2.11. Preparation of Total/Nuclear Lysates of Cells

B16F10 cells were treated with *α*-MSH (100 nM), Pc-EE (0, 50, and 100 *μ*g/mL), or arbutin (1 mM) for 48 h. To prepare whole lysates, cells were collected with trypsin, washed with cold 1x PBS, and lysed in lysis buffer (50 mM Tris-HCL, pH 7.5, 20 mM NaF, 25 mM *β*-glycerol phosphate, pH 7.5, 120 mM NaCl, 2% NP-40, 2 *μ*g/mL leupeptin, 2 *μ*g/mL aprotinin, 2 *μ*g/mL pepstatin A, 100 *μ*M Na_3_VO_4_, 1 mM benzamide, 100 *μ*M PMSF, and 1.6 mM pervanadate) for 30 min with rotation at 4°C. The lysates were used after clarified by centrifugation at 16,000g for 10 min at 4°C. To prepare nuclear lysates, we conducted a three-step procedure. First, cells were collected with trypsin, washed with cold 1x PBS, treated with buffer A (20 mM Tris-HCL, pH 8.0, 10 mM EGTA, 2 mM EDTA, 2 mM DTT, 1 mM PMSF, 25 *μ*g/mL aprotinin, and 10 *μ*g/mL leupeptin), and sonicated for 10 s at output 4. After sonication, nuclear lysates were collected by centrifugation at 7,000g for 15 min at 4°C. In the second step, the pellet (the nuclear fraction) was washed once in buffer A. After washing, we treated it with buffer B (buffer A added 1% Triton X-100). And then, we performed sonication for 10 s at output 4. The nuclear lysates were used after clarified by centrifugation at 16,000g for 10 min at 4°C.

### 2.12. Immunoblotting

The lysates (total/nuclear) prepared from B16F10 cells were subjected to western blot analysis for the amounts of the total and phosphoforms of tyrosinase, CREB, MITF, lamin A/C, JNK, ERK, p38, LC3B, and *β*-actin. Each antibody was reacted with 3% FBS in TBST for 2 h at a ratio of 1 : 2,500. After 2 h, each of the second antibodies (rabbit or mouse) was reacted at a ratio of 1 : 2,500 for 2 h to form a band. Immunoreactive bands were visualized as described previously [23].

### 2.13. Confocal Microscopy

HEK293T cells (2.0 × 10^5^ cells/well in 12-well plates) were transfected with 1 *μ*g/mL of plasmids driving the expression of GFP-LC3B using the polyethylenimine (PEI) method. After incubation for 24 h, the cells were treated with either 100 *μ*g/mL Pc-EE or vehicle. For confocal microscopy, HEK293T cells were fixed in 4% paraformaldehyde (PFA) in PBS for 10 min, followed by membrane permeabilization solution (0.5% Triton X-100) for 10 min. The cells were then blocked with 1% bovine serum albumin (BSA) in PBS for 1 h at room temperature, followed by incubation overnight at 4^o^C with anti-LC3B antibodies. The cells were then treated with Alexa Fluor 488-conjugated secondary antibodies for 1 h at room temperature. For DNA counterstaining, 10 *μ*g/mL Hoechst 33342 in PBS was applied for 30 min. PBS washing was performed twice in each step for 5 min. After mounting the cells on glass slides, they were imaged using a laser-scanning confocal microscope (Zeiss LSM 710 META, Oberkochen, Germany) with a 63x oil-immersion objective lens.

### 2.14. Statistical Analysis

All data are presented as mean ± standard deviation, and each experiment consisted of three to four replications. The Mann–Whitney *U* test was used to analyze the statistical differences between groups. A *p* value < 0.05 was regarded as statistically significant. All statistical tests were performed using SPSS software, version 22.0 (IBM Corp., Armonk, NY, USA).

## 3. Results

### 3.1. Effects of Pc-EE on the Viability of B16F10 and HEK293T Cells and HPLC Analysis of Pc-EE

**Figure 1:**
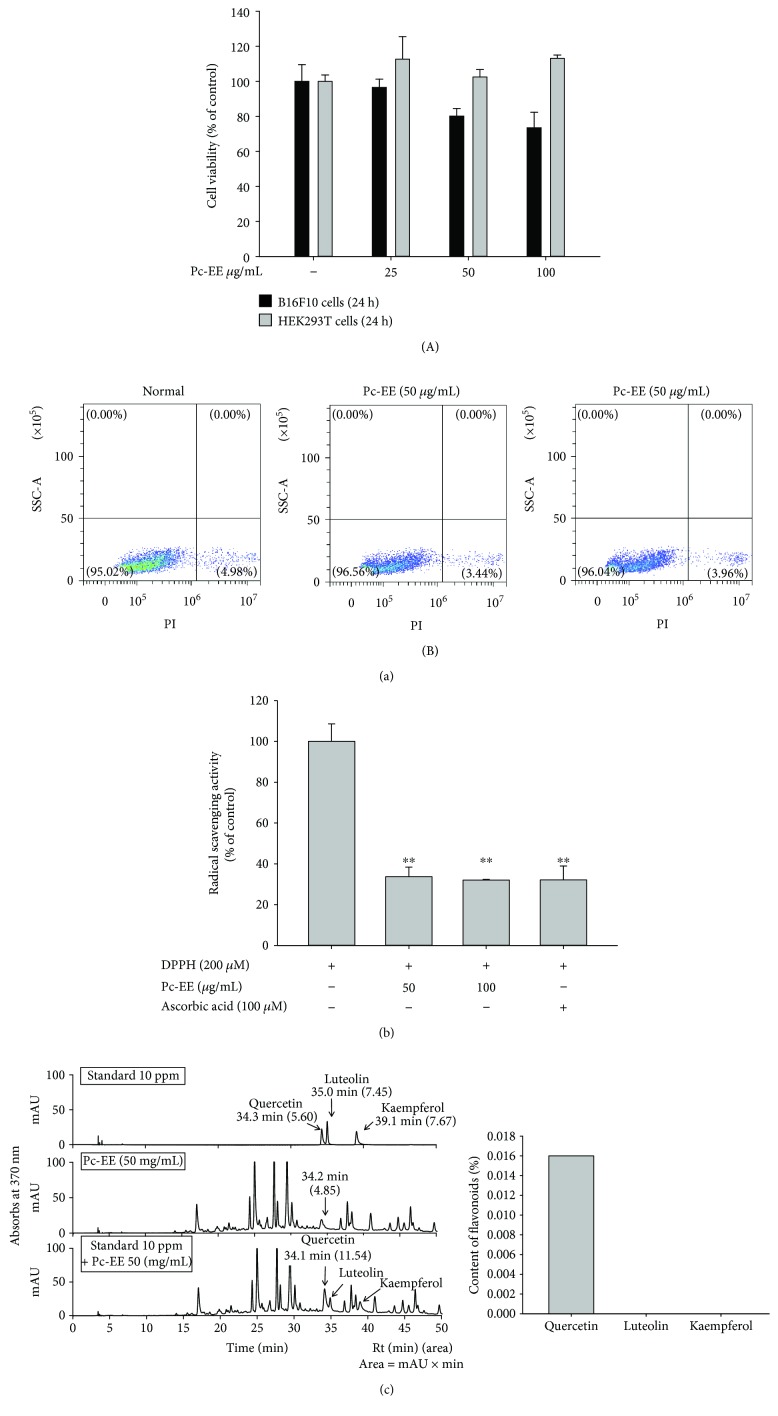
Effects of Pc-EE on the viability of B16F10 and HEK293T cells and HPLC analysis of Pc-EE extract. (a1, a2) B16F10 and HEK293T cells were incubated with various concentrations (0 to 100 *μ*g/mL) of Pc-EE for 24 h. Cell viability was determined using the MTT assay (a1) and PI staining (a2). (b) Cells were cotreated with DPPH and either various concentrations of Pc-EE (0 to 100 *μ*g/mL) or 100 *μ*M ascorbic acid. (c) The phytochemical profile of Pc-EE (10 *μ*L of 50 mg/mL) was analyzed using HPLC with standard compounds (quercetin, luteolin, and kaempferol). ^∗∗^ *p* < 0.01 and ^∗^ *p* < 0.05 compared to the control group.

To confirm the toxicity of Pc-EE, we examined cell viability using HEK293T and B16F10 cells. As shown in Figure 1(a1), Pc-EE was not cytotoxic at concentrations up to 100 *μ*g/mL in HEK293T cells but B16F10 cells exhibited 73% survival at 100 *μ*g/mL of Pc-EE. However, there was no significant difference between treatment of this extract and the vehicle-treated group in HaCaT cells according to the PI-staining experiment to show late apoptotic cells (Figure 1(a2)), implying weak or no cytotoxic activity of Pc-EE. Recent studies have shown that Pc-EE has a strong antioxidant effect, which we confirmed using DPPH assays [16, 17]. As shown in Figure 1(b), Pc-EE showed similar antioxidant activity to ascorbic acid, the positive control, showing 67%. To determine the phytochemical properties of Pc-EE, the flavonoid content was measured using HPLC with three flavonoid standards (quercetin, luteolin, and kaempferol). As shown in Figure 1(c), quercetin but not luteolin and kaempferol was detected up to 0.016% in 50 mg/mL of Pc-EE.

### 3.2. Effects of Pc-EE against UVB- or H_2_O_2_-Induced Cell Death, Collagen Degradation, Inflammatory Response, Moisture Loss, and Oxidation in HaCaT Cells

**Figure 2:**
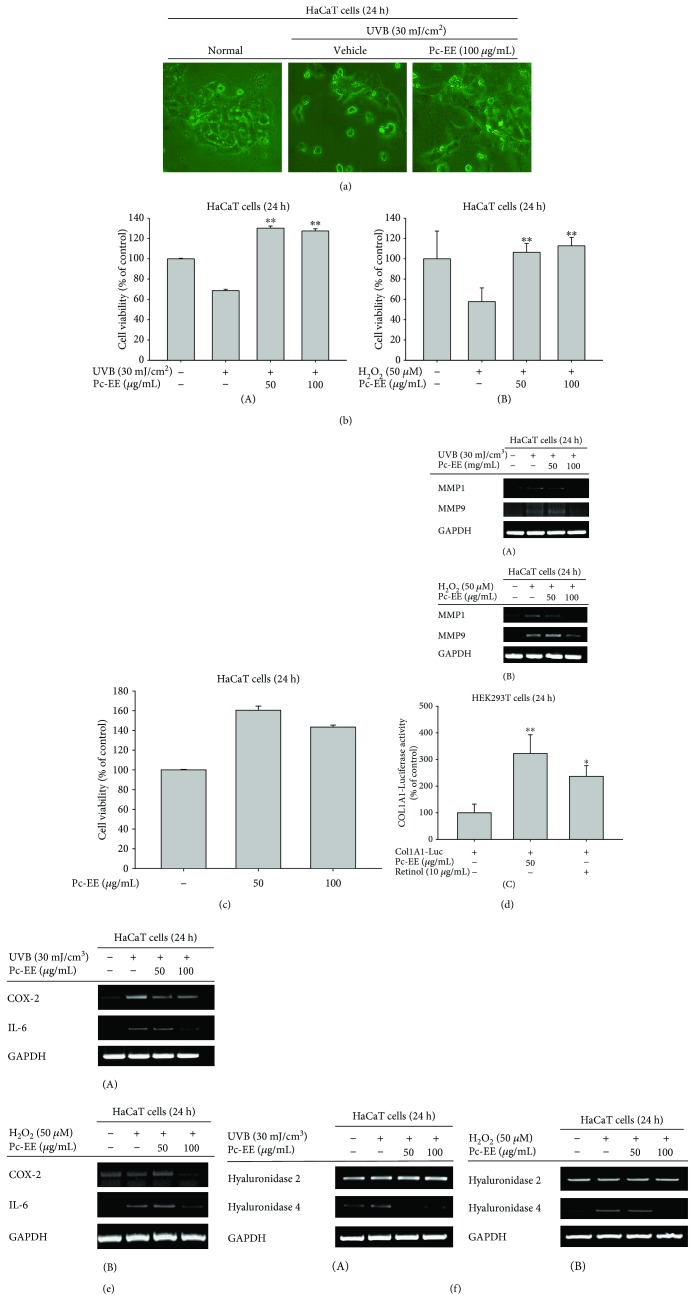
Effects of Pc-EE against UVB- or H_2_O_2_-induced cell death, collagen degradation, inflammatory response, moisture loss, and oxidation in HaCaT cells. (a) HaCaT cells, with and without UVB irradiation (30 mJ/cm^2^), were treated with 100 *μ*g/mL Pc-EE for 24 h. Cell morphology was determined via microscopy. (b1–c) HaCaT cells, with or without UVB irradiation (30 mJ/cm^2^) and H_2_O_2_ (50 *μ*M), were treated with various concentrations of Pc-EE (0 to 100 *μ*g/mL) for 24 h. Cell viability was determined using the MTT assay. (d1–f2) HaCaT cells were exposed to either UVB irradiation (30 mJ/cm^2^) or H_2_O_2_ (50 *μ*M) and treated with Pc-EE (50 or 100 *μ*g/mL) for 24 h. The mRNA levels of inflammatory genes and moisturizing factors were then determined using RT-PCR. (d3) The promoter binding activity of the transcription factor Col1A1 was analyzed using a reporter gene assay. HEK293T cells were transfected with plasmids driving the expression of Col1A1-Luc (1 *μ*g/mL) and *β*-gal (transfection control). The cells were then treated with or without 50 *μ*g/mL Pc-EE or 10 *μ*g/mL retinol for 24 h. Luciferase activity was measured using a luminometer. ^∗∗^ *p* < 0.01 and ^∗^ *p* < 0.05 compared to the control (UVB or H_2_O_2_ alone (d1–d3) or Col1A-Luc alone (d3)) group.

To confirm the protective effects of Pc-EE against UVB irradiation-induced damage and oxidative stress in HaCaT cells, we performed cytomorphological analysis and MTT assays. As shown by the cytomorphological analysis in Figure 2(a), UVB irradiation induced cell death, which was decreased by 100 *μ*g/mL Pc-EE. Additionally, the MTT assays revealed that cell viability was increased by Pc-EE (50 or 100 *μ*g/mL) following UVB irradiation or H_2_O_2_ treatment (Figures 2(b1) and 2(b2)) up to 100-130% from 60-70%. In addition, Pc-EE alone was not cytotoxic at concentrations up to 100 *μ*g/mL (Figure 2(c)). These data indicate that Pc-EE inhibits apoptosis induced by UVB irradiation or oxidative stress.

UVB irradiation or oxidative stress can induce expression of matrix metalloproteinases (MMPs), which are proteins, such as collagens, that degrade extracellular matrix proteins, resulting in wrinkles [1]. To evaluate the inhibitory effects of Pc-EE on MMP production, we performed reverse transcription-polymerase chain reaction (RT-PCR) assays under UVB irradiation or H_2_O_2_-treatment conditions using HaCaT cells. Both UVB- and H_2_O_2_-induced matrix metalloproteinase-1 (MMP-1) mRNA levels were dose-dependently reduced by Pc-EE, and MMP9 mRNA expression was inhibited by 100 *μ*g/mL Pc-EE (Figures 2(d1) and 2(d2)). In addition, luciferase reporter analysis using a plasmid containing the binding site for the Col1A1 collagen synthesis promoter resulted in increase of luciferase activity up to 317% at 50 *μ*g/mL Pc-EE. The positive control, retinol (10 *μ*g/mL), increased luciferase activity 2.5 times (Figure 2(d3)). These data indicate that Pc-EE can inhibit MMP expression and induce collagen synthesis to inhibit wrinkle formation caused by UVB irradiation or oxidative stress.

UVB irradiation induces oxidative stress by creating reactive oxygen species (ROS) [1], which cause an inflammatory reaction in keratinocytes [2]. To confirm the effects of Pc-EE on inflammatory responses induced by UVB irradiation or oxidative stress, we determined the expression levels of inflammation-related mRNA using RT-PCR assays. As shown in Figures 2(e1) and 2(e2), the expression of mRNA from the inflammatory factors cyclooxygenease-2 (COX-2) and interleukin-6 (IL-6) mRNA was suppressed at 100 *μ*g/mL Pc-EE. These data indicate that Pc-EE inhibits the inflammatory response caused by UVB irradiation or H_2_O_2_.

Oxidative stress induces hyaluronidase expression and degrades hyaluronic acid (HA) [11, 12]. As shown in Figures 2(f1) and 2(f2), 100 *μ*g/mL Pc-EE only inhibited the expression of hyaluronidase 4 mRNA under UV irradiation- or H_2_O_2_-treatment conditions; hyaluronidase 2 mRNA expression was not inhibited. This indicates that Pc-EE can produce a moisturizing effect by inhibiting the hyaluronidase 4 mRNA expression. Taken together, these data indicate that Pc-EE exhibits antiaging effects by inhibiting oxidative stress induced by UVB irradiation or H_2_O_2_.

### 3.3. Antimelanogenic Effects of Pc-EE in *α*-MSH-Treated B16F10 Cells

**Figure 3:**
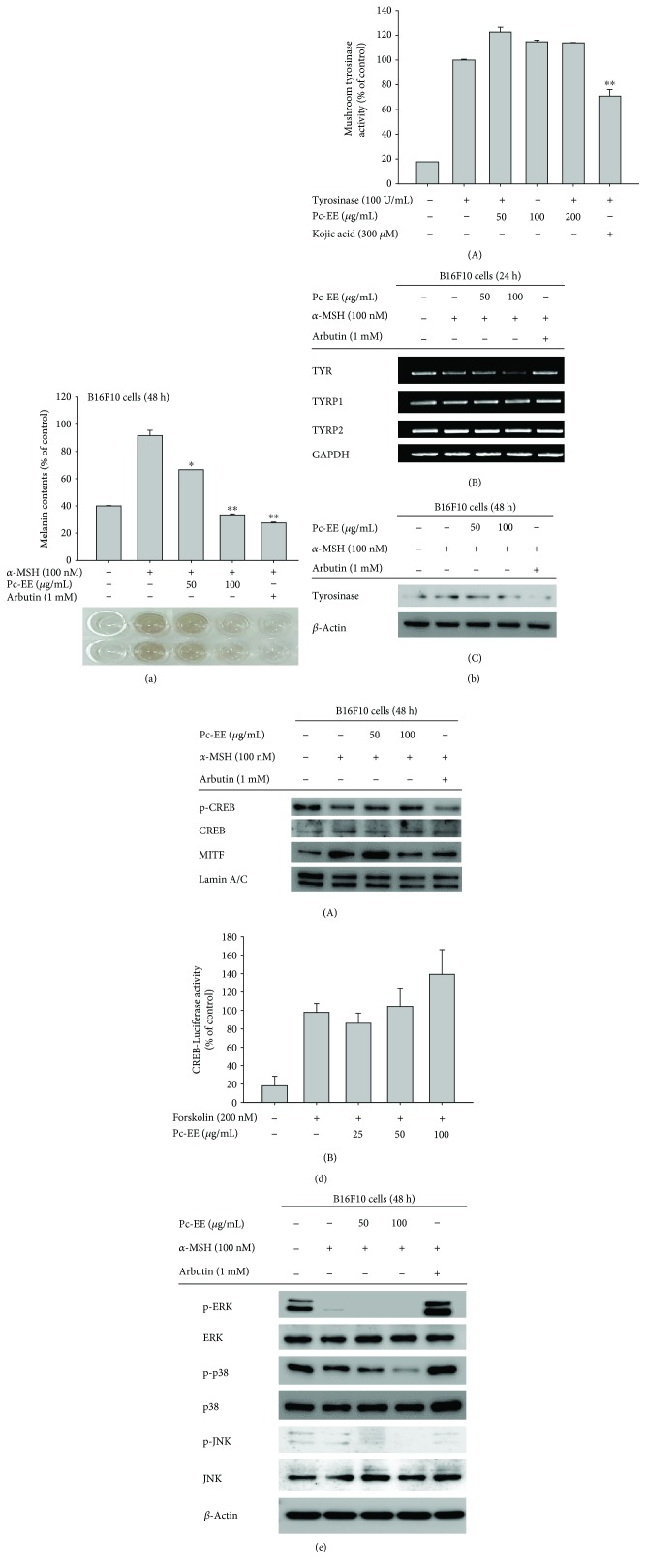
Antimelanogenic effects of Pc-EE in *α*-MSH-treated B16F10 cells. (a) B16F10 cells were treated with *α*-MSH (100 nM) in the presence or absence of Pc-EE (50 and 100 *μ*g/mL) or arbutin (1 mM) for 48 h, and the melanin level was determined. (b1) The effects of Pc-EE (50 to 200 *μ*g/mL) or kojic acid (300 *μ*M) on mushroom tyrosinase activity were determined by quantifying the activity of purified tyrosinase. (b2) B16F10 cells were cotreated with *α*-MSH (100 nM) and either Pc-EE (50 or 100 *μ*g/mL) or arbutin (1 mM) for 24 h. The mRNA levels of tyrosinase-related genes were then determined using RT-PCR. (b2, c1, and d) The levels of phosphorylated and total tyrosinase, CREB, MITF, ERK, p38, JNK, and *β*-actin proteins in B16F10 cells were determined using either phospho-specific or total antibodies for each protein. (c2) The promoter binding activity of the transcription factor CREB was analyzed using a reporter gene assay. HEK293T cells were transfected with plasmids driving the expression of CREB-Luc (1 *μ*g/mL) and *β*-gal (transfection control). The cells were then cotreated with either forskolin (200 nM) or Pc-EE (25 to 50 *μ*g/mL) for 24 h. Luciferase activity was measured using a luminometer. ^∗∗^ *p* < 0.01 and ^∗^ *p* < 0.05 compared to the control (*α*-MSH (a) or tyrosinase alone (b1)) group.

To examine the effects of Pc-EE on melanin production, B16F10 melanoma cells were treated with the melanin inducer *α*-MSH. Pc-EE lowered the melanin content in a dose-dependent manner up to the basal level (38%), comparable with that of the melamine-production inhibitor arbutin (Figure 3(a)). Because tyrosinase is a key enzyme in melanogenesis [24], we determined whether Pc-EE regulates either the activity or the transcription level of tyrosinase. In the mushroom tyrosinase activity assay, the inhibitory effect of the tyrosinase inhibitor kojic acid (300 *μ*M) was 30%, but Pc-EE at concentrations from 50 to 200 *μ*g/mL exhibited no inhibition (Figure 3(b1)). However, mRNA analysis revealed a decrease in the mRNA level of TYR by Pc-EE in a concentration-dependent manner at 24 h. However, the mRNA levels of TYRP1 and TYRP2 were not decreased by Pc-EE (Figure 3(b2)). In addition, the protein level of tyrosinase was decreased by Pc-EE in a concentration-dependent manner (Figure 3(b3)). These data indicate that Pc-EE has an inhibitory effect on tyrosinase.

To confirm the mechanism of Pc-EE inhibition of melanogenesis, we studied the activity of MITF, which modulates the TYR mRNA level through transcription factors [7, 11, 13]. In the nuclear protein fraction, the level of MITF was decreased at 100 *μ*g/mL Pc-EE. However, the protein level of CREB, a transcription factor of MITF, was not decreased (Figure 3(c1)). Melanin production is ultimately regulated by a variety of signaling pathways leading to MITF expression and activation. Among them, the CREB signaling pathway is activated by *α*-MSH/MC1R signaling [25]. To determine the effects of Pc-EE on *α*-MSH/MC1R signaling in melanogenesis, luciferase reporter assays using plasmids containing CREB binding sites were performed. As expected, CREB-luciferase activity was increased by the inducer forskolin (200 nM), but treatment with Pc-EE did not reduce CREB-luciferase activity (Figure 3(c2)). These data indicate that, in melanogenesis, Pc-EE can inhibit MITF activity. Since MITF activity and inhibition are modulated by MAPKs [26], we assessed the effects of Pc-EE on the activity of MAPKs. Although B16F10 melanoma cells are activated by MAPKs [27, 28], the activity of phospho-ERK, which inhibits melanin production, was reduced due to stimulation by *α*-MSH. However, Pc-EE further reduced the phospho-ERK level. In contrast, the levels of activated phospho-p38 and phospho-JNK were inhibited by Pc-EE at 100 *μ*g/mL (Figure 3(d)). These data indicate that Pc-EE regulates MAPK activity, such as p38 and JNK, and modulates melanogenesis by regulating MITF activity.

### 3.4. Effects of Pc-EE on the Autophagy Signaling Pathway

**Figure 4:**
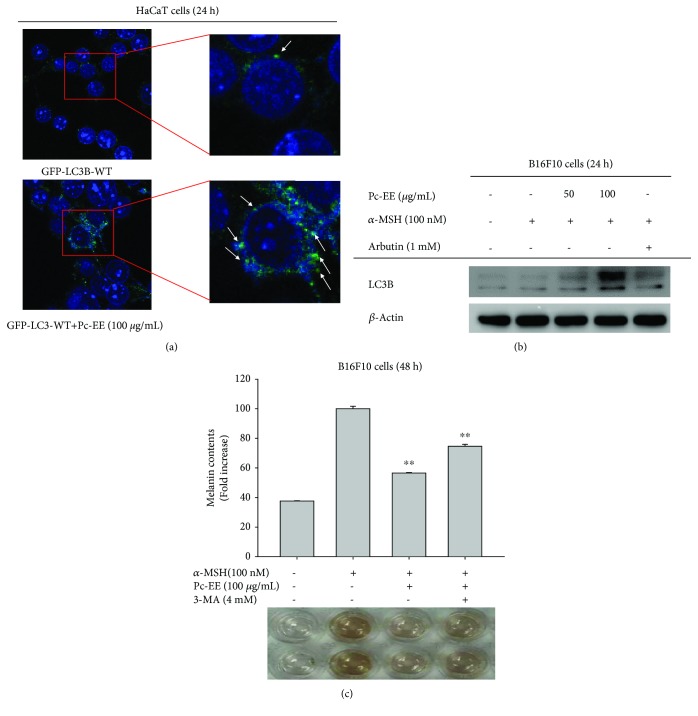
Effects of Pc-EE on the autophagy signaling pathway. (a) HEK293T cells were transfected with plasmids driving the expression of GFP-LC3-WT (1.8 *μ*g/mL) and treated with 100 *μ*g/mL Pc-EE. Confocal microscopy images (63x) were acquired using a laser-scanning confocal microscope (Zeiss LSM 710 META). (b) The levels of total LC3B and *β*-actin proteins were determined in B16F10 cells using antibodies for each protein. (c) B16F10 cells were treated with *α*-MSH (100 nM) in the presence or absence of Pc-EE (50 and 100 *μ*g/mL) or 3-methyladenine (4 mM) for 48 h, and the melanin level was determined. ^∗∗^ *p* < 0.01 and ^∗^ *p* < 0.05 compared to the control (*α*-MSH alone (c)) group.

To confirm the effects of Pc-EE on the regulation of autophagy activity during melanin production, we measured the expression of LC3B, a maker of autophagy. HEK293T cells were transfected with the GFP-LC3B plasmid and treated with Pc-EE (100 *μ*g/mL). As determined using confocal microscopy, the level of GFP-LC3B expression was increased by 100 *μ*g/mL Pc-EE (Figure 4(a)). Also, as determined using immunoblotting, the level of LC3B protein was increased at a concentration of 100 *μ*g/mL Pc-EE in B16F10 cells (Figure 4(b)). These data indicate that Pc-EE can induce autophagy in B16F10 and HEK293T cells. Melanin content was also investigated using the autophagy inhibitor 3-methyladenine (3-MA). Treatment with *α*-MSH increased the melanin content, which was decreased approximately by 55% during treatment of Pc-EE (100 *μ*g/mL). However, the content of melanin was restored to approximately 80% by 3-MA treatment (Figure 4(c)). These data show that Pc-EE induces autophagy, which then inhibits melanogenesis.

## 4. Discussion

*Penthorum chinense* Pursh has traditionally been used in China for the treatment of jaundice, cholecystitis, edema, and infectious hepatitis [16]. *Penthorum chinense* Pursh is distributed not only in China but also in Korea and Japan and is used as a medicinal plant or tea. Recently, biological studies using *Penthorum chinense* Pursh have shown that it has antihepatocarcinoma [29] and hepatoprotective effects [16, 30], as well as excellent antioxidant effects [17, 31, 32]. However, no studies have reported on the effects of *Penthorum chinense* Pursh on UVB irradiation- or H_2_O_2_-induced oxidative stress in skin cells. Therefore, this study focused on the effects of Pc-EE on the underlying molecular mechanisms of UVB irradiation and oxidative stress in skin cells.

First, the viability of B16F10, HEK293T, and HaCaT cells was analyzed after exposure to Pc-EE to determine the range of noncytotoxic doses of *Penthorum chinense* Pursh ethanol extract (Pc-EE). As shown in Figures 1(a1) and 1(a2) and Figure 2(c), Pc-EE extract showed no significant inhibition of cell viability up to 100 *μ*g/mL in HEK293T and HaCaT, but it reduced the viability of B16F10 cells by approximately 20%. Therefore, 100 *μ*g/mL Pc-EE was used for subsequent experiments. As shown in Figure 1(b), Pc-EE significantly inhibited radical scavenging activity compared with ascorbic acid, the control antioxidant. To determine the phytochemical properties of Pc-EE, the flavonoid content was analyzed using the standards quercetin, luteolin, and kaempferol. As shown in Figure 1(c), quercetin was detected as a major flavonoid of Pc-EE but luteolin and kaempferol were not detected.

Skin aging is caused by various factors, such as UV irradiation and stress, and wrinkle formation due to photoaging is related to oxidative stress and inflammatory reactions [33]. Of those factors, UVB irradiation causes oxidative stress in skin cells, resulting in the death of skin cells [1], as well as biological phenomena such as increased MMP [1], increased inflammatory mediators [2], and decreased collagen synthesis. Therefore, we determined the effects of Pc-EE on various biological phenomena under oxidative stress conditions caused by UV irradiation or H_2_O_2_. First, we investigated the effects of Pc-EE against oxidative stress-induced cell death caused by UVB irradiation or H_2_O_2_ treatment in keratinocyte. As shown in Figure 2(a–b2), oxidative stress induced by UVB irradiation or H_2_O_2_ was cytotoxic to keratinocytes and decreased their viability but the cytotoxicity was ameliorated by Pc-EE. In addition, expression of inflammatory mediators (COX-2 and IL-6 mRNA) and matrix metalloproteinases (MMP1 and MMP9 mRNA) was inhibited by Pc-EE. In the luciferase reporter analysis using a plasmid containing the binding site for the Col1A1 collagen synthesis promoter, treatment with Pc-EE resulted in a 300% increase in luciferase activity (Figure 2(d1–e2)). Taken together, these data demonstrate that Pc-EE protects skin cells through anti-inflammatory effects and inhibition of MMPs and inhibits wrinkles by inducing collagen synthesis. Hyaluronic acid (HA) is an integral part of the extracellular matrix of basal keratinocytes [9] and performs a key role in the epidermis by maintaining the extracellular space and providing a hydrated structure for delivery of nutrients [10]. Oxidative stress induces hyaluronidase, an enzyme that degrades HA [34]. To confirm the effect of Pc-EE on the degradation of HA by oxidative stress, the expression level of hyaluronidase mRNA was determined using RT-PCR. As shown in Figures 2(f1) and 2(f2), UVB irradiation- and H_2_O_2_-induced hyaluronidase 4 expression was inhibited by 100 *μ*g/mL of Pc-EE but hyaluronidase 2 expression was not inhibited under the same conditions. As shown in the suppression of MMP expression, inhibition of inflammation-regulatory genes increased under exposure of UVB, and H_2_O_2_ was shown during the Pc-EE-treated state. These data show that the potent antioxidant effect of Pc-EE modulates the skin aging response to UVB irradiation- or H_2_O_2_-induced oxidative stress in keratinocytes.

UV irradiation induces the activation of p53 in keratinocytes, and activated p53 upregulates proopiomelanocortin (POMC). After translation, POMC is cleaved to produce beta-endorphin and *α*-MSH, which binds to MC1R in melanocytes, activating signal transduction to induce melanin synthesis [3]. To investigate the effects of Pc-EE on melanin production, we activated the B16F10 melanoma cell line with *α*-MSH and treated them with Pc-EE. As shown Figure 3(a), Pc-EE strongly decreased the melanin content. The binding of *α*-MSH and MC1R activates the protein kinase A (PKA) pathway, increasing the phosphorylation of the cAMP response element binding (CREB) protein, which enters the nucleus to induce microphthalmia-associated transcription factor (MITF). MITF is regulated by MAPKs, and activated MITF increases the gene expression of tyrosinase, TYRP1, and TYRP2, the main enzymes involved in melanogenesis [13]. We therefore studied each step of the melanin production to determine the mechanism of Pc-EE inhibition of melanin production. As shown in Figure 3(b1–b3), Pc-EE did not directly inhibit tyrosinase activity but tyrosinase mRNA and protein levels were significantly reduced by Pc-EE. The mitogen-activated protein kinase (MAPK) pathway is tightly regulated in normal melanocytes but abnormally regulated in melanoma cells [27, 28]. For this reason, mRNA and proteins of MAPK were overexpressed in the group not treated with *α*-MSH [27, 28]. To confirm the activity of the transcription factor that expresses tyrosinase, the level of MITF in the nucleus was evaluated. As shown Figure 3(c1), the level of MITF in the nucleus was reduced by Pc-EE, although the level of phospho-CREB, a transcription factor that induces MITF expression, was not changed. Therefore, it was confirmed that Pc-EE does not inhibit the CREB signaling pathway but acts in the signal transduction step to activate MITF. These results were confirmed using luciferase reporter analysis with a plasmid containing the binding site for CREB (Figure 3(c2)). The evaluation of Pc-EE's effect on MAPKs showed that phospho-p38 and phospho-JNK levels were significantly inhibited at a concentration of 100 *μ*g/mL Pc-EE. However, the activity of ERK in inhibiting the activity of MITF remained reduced despite treatment with Pc-EE (Figure 3(d)). Recently, it has been reported that autophagy plays an important role in melanogenic regulation and that induction of autophagy activity inhibits melanin production [35, 36]. Furthermore, autophagy is activated by the flavonoid quercetin [37]. We determined whether Pc-EE regulates melanogenesis through autophagy by determining the activity of the autophagy marker LC3B. As shown in Figures 4(a) and 4(b), Pc-EE stimulated the activity of LC3B and induced autophagy. To confirm that autophagy induced by Pc-EE regulates melanogenesis, autophagy inhibitors were studied in relation to melanin content. As shown in Figure 4(c), the melanin content increased approximately 20% after treatment with 3-MA, which is an autophagy inhibitor, implying that 20% of the recovery effect by autophagy inhibition is thought to be the result of MAPK regulation in Pc-EE. However, further detailed study should be followed to clearly verify this.

**Figure 5:**
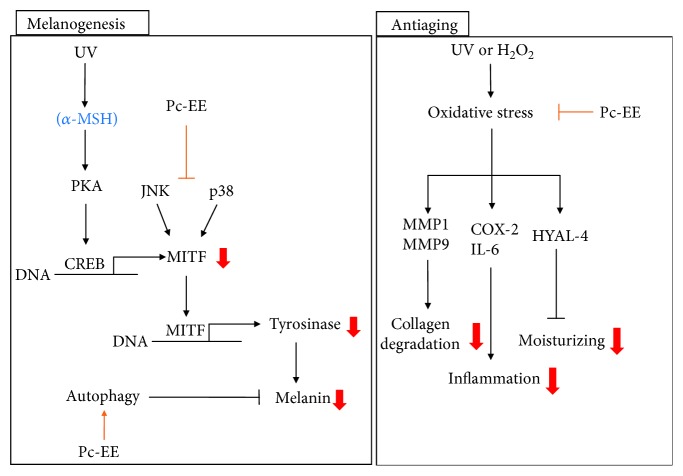
Putative inhibitory pathway of Pc-EE.

In summary, we demonstrated that Pc-EE exerts antioxidant effects to protect against factors of skin aging, such as skin cell death, MMP expression, inflammatory gene expression, and water loss, under UVB irradiation- or H_2_O_2_-induced oxidative stress conditions. As mentioned earlier, the antioxidant effect of Pc-EE is known to have antihepatocellular- and liver-protective effects. However, in this study, we found that the antioxidant effect of Pc-EE can contribute to skin health through inhibition of oxidative stress. In addition, we showed a novel inhibitory effect of Pc-EE on melanin formation by inducing autophagy and controlling MAPKs in melanocytes (Figure 5). Quercetin, which was detected in the analysis of Pc-EE phytochemical properties (Figure 1(b)), has antioxidant, anti-inflammatory [38], antimelanogenic [39], and autophagy-inducing effects [37] and appears to play a major role in the efficacy of Pc-EE in this study. Thus, this study strongly suggests that Pc-EE blocks the photoaging process and inhibits melanogenesis, demonstrating the potential use of drugs or cosmetics containing Pc-EE.

## Figures and Tables

**Table 1:** PCR primers used in this study.

Gene name		Sequence (5′ to 3′)
*Human*		
MMP1	F	TCTGACGTTGATCCCAGAGAGCAG
	R	CAGGGTGACACCAGTGACTGCAC
MMP9	F	GCCACTTGTCGGCGATAAGG
COX-2	R	CACTGTCCACCCCTCAGAGC
	F	GGGATTTTGGAACGTTGTGAA
	R	CGACATTGTAAGTTGGTGGACTGT
IL-6	F	TACCCCCAGGAGAAGATTCC
	R	TTTTCTGCCAGTGCCTCTTT
Hyaluronidase 2	F	TACACCACAAGCACGGAGAC
Hyaluronidase 4	R	ATGCAGGAAGGTACTGGCAC
	F	TGAGCTCTCTTGGCTCTGGA
	R	AGGCAGCACTTTCTCCTATGG
GAPDH	F	GGTCACCAGGGCTGCTTTTA
	R	GATGGCATGGACTGTGGTCA
*Mouse*		
TYR	F	GTCCACTCACAGGGATAGCAG
	R	AGAGTCTCTGTTATGGCCGA
TYRP1	F	ATGGAACGGGAGGACAAACC
	R	TCCTGACCTGGCCATTGAAC
TYRP2	F	CAGTTTCCCCGAGTCTGCAT
	R	GTCTAAGGCGCCCAAGAACT
GAPDH	F	ACCACAGTCCATGCCATCAC
	R	CCACCACCCTGTTGCTGTAG

## Data Availability

The data used to support the findings of this study are available from the corresponding authors upon request.

## Abbreviations

Pc-EE: *P. chinense Pursh* ethanol extract
MMPs: Matrix metalloproteinases
COX-2: Cyclooxygenease-2
IL-6: Interleukin-6
MITF: Microphthalmia-associated transcription factor
*α*-MSH: *α*-Melanocyte-stimulating hormone
3-MA: 3-Methyladenine
TYRP: Tyrosinase-related protein
UV: Ultraviolet
CREB: cAMP response elements (CRE)
MC1R: Melanocortin 1 receptor
HA: Hyaluronic acid
RT-PCR: Reverse transcription-polymerase chain reaction
MAPKs: Mitogen-activated protein kinases
MTT: 3-(4,5-Dimethylthiazol-2-yl)-2,5-diphenyltetrazolium bromide.
